# C-855-07. Frailty as a Predictor of In-hospital and Long-Term Mortality in Elderly Burn Patients

**DOI:** 10.1093/jbcr/irag033.091

**Published:** 2026-04-06

**Authors:** Deepak K Ozhathil, Rachel Schneider, Jane Boyle, Colette Galet, Nao Mimoto, Lucy Wibbenmeyer, Kathleen S Romanowski, Shawn Tejiram, David M Hill, Alisa Savetamal, Dhaval Bhavsar, Kim Priban, Rosemary E Paine, Sam Miotke, Sara Higginson, Theresa L Chin, Alexandra M Lacey

**Affiliations:** Akron Children's Hospital, Akron, OH; Cleveland Clinic Foundation, Cleveland, OH; Northeast Ohio Medical University, Orange, OH; University of Iowa, Iowa City, IA; The University of Akron, Akron, OH; University of Iowa Health Care, Iowa City, IA; UC Davis Burn Center & Shriners Hospital for Children, Sacramento, CA; MedStar Washington Hospital Center, Washington, DC; Firefighters Regional One Burn Center, Memphis, TN; Bridgeport Hospital/Yale New Haven Health, Bridgeport, CT; The University of Kansas Health System, Kansas City, KS; Akron Children's Hospital, Akron, OH; MaineHealth Medical Center, Portland, ME; Regions Hospital, Saint Paul, MN; Shriners Children's - Ohio, Dayton, OH; University of California Irvine, Orange, CA; Regions Hospital, Saint Paul, MN

## Abstract

**Introduction:**

Older adults face disproportionately high morbidity and mortality after burn injury due to altered tissue composition, reduced mobility, cognitive decline, and sensory impairment. The modified Baux score - a composite measure of age, total body surface area (TBSA) burned, and inhalation injury - remains the most utilized model for acute burn mortality risk prediction. Frailty, however, has emerged as a significant determinant of adverse outcomes and has been used to discuss ICU utilization, prolonged ventilation, non-home discharge, and mortality risk. Despite its broad application, frailty has not been systematically examined as a mortality risk factor following burn injury. This study is the first to evaluate frailty in the context of established burn mortality models, with the goal of determining its predictive value for both in-hospital and long-term survival.

**Methods:**

A multicenter consortium of 12 burn centers compiled a retrospective dataset of patients aged ≥60 years admitted for burn injury. A total of 1632 records were available, incorporating 156 demographic and clinical variables, as well as Canadian Study of Health and Aging–Clinical Frailty Scale (CFS) scores. Records missing frailty or mortality data were excluded. The primary outcomes were in-hospital mortality (IHM) and long-term mortality (LTM), defined as all-cause death within three years of injury. Predictive performance was assessed using area under the curve (AUC) modeling, trained on 80% of the dataset and validated on the remaining 20%.

**Results:**

A total of 1209 patients were included for IHM models and 915 for LTM models. CFS was an independent predictor of IHM (AUC 62.6%; *p*=.000003) compared to the Baux score (90.2%). When combined, frailty improved prediction accuracy for IHM (AUC 91.8%; *p* = < 0.000001). The largest mortality risk increase was observed at CFS ≥4 (IHM AUC 60.3%, *p*=.00001; LTM AUC 69.7%, *p* = < 0.000001). For LTM, frailty outperformed Baux (76.2% vs. 51.8%), underscoring its importance beyond the acute phase.

**Conclusions:**

Frailty, particularly CFS ≥4, significantly augments mortality prediction in older adult burn survivors, complementing the established modified Baux score for in-hospital outcomes and providing superior prognostic value for long-term survival.

**Applicability of Research to Practice:**

These findings support routine incorporation of frailty into burn mortality prediction models. Early identification of frailty may improve risk stratification, guide resource allocation, and provide the foundation for future prospective studies.

**Funding for the study:**

N/A.

